# Risk Factors Affecting the Severity of Full-Term Neonatal Retinal Hemorrhage

**DOI:** 10.1155/2017/4231489

**Published:** 2017-07-19

**Authors:** Zhang Yanli, Zhao Qi, Lin Yu, Guo Haike

**Affiliations:** ^1^Southern Medical University, Guangzhou, Guangdong 510515, China; ^2^Guangdong General Hospital and Guangdong Academy of Medical Sciences, Guangzhou, Guangdong 510080, China; ^3^Department of Ophthalmology, Zhongshan City People's Hospital, Zhongshan Affiliated Hospital of Sun Yat-sen University, Zhongshan, Guangdong 528403, China

## Abstract

**Objective:**

The purpose of this study was to explore the underlying clinical factors associated with the degree of retinal hemorrhage (RH) in full-term newborns.

**Methods:**

A total of 3054 full-term infants were included in this study. Eye examinations were performed with RetCamIII within one week of birth for all infants. Maternal, obstetric, and neonatal parameters were compared between newborns with RH and controls. The RH group was divided into three sections (I, II, and III) based on the degree of RH.

**Results:**

RH was observed in 1202 of 3054 infants (39.36%) in this study. The quantity and proportion of newborns in groups I, II, and III were 408 (13.36%), 610 (19.97%), and 184 (6.03%), respectively. Spontaneous vaginal delivery (SVD), prolonged duration of second stage of labor, advanced maternal age, and neonatal intracranial hemorrhage positively correlated with aggravation of the degree of RH in newborns. Conversely, cesarean section was protective against the incidence of RH.

**Conclusions:**

SVD, prolonged duration of second stage of labor, advanced maternal age, and neonatal intracranial hemorrhage were potential risk factors for aggravation of the degree of RH in full-term infants. Accordingly, infants with these risk factors may require greater attention with respect to RH development.

## 1. Introduction

Neonatal retinal hemorrhage (RH) is a clinically common neonatal fundus condition occurring in newborns within one month of birth [1]. In general, neonatal RH does not affect the development of visual function, although macular hemorrhage may lead to amblyopia due to the delayed absorption caused by bleeding [2]. The prognosis is often poor in clinically severe RH, and the quality of life of the affected child is likely to be seriously affected. Presently, the risk factors and mechanisms underlying neonatal RH are not well-understood. While there are clinical studies reporting risk factor analyses of RH, there are no known systematic investigations of the correlation between the various risk factors and the degree of RH. To investigate risk factors associated with increases in RH for newborns, we performed a statistical analysis of the incidence and degree of RH in a large sample of full-term infants.

## 2. Methods

### 2.1. Subjects

Three thousand and fifty-four full-term infants born at the Affiliated Zhongshan Hospital of Sun Yat-sen University (Zhongshan City People's Hospital) between January 2013 and December 2014 were included in the study. All infants had a gestational age of more than 37 weeks and Apgar score of nine or above; they underwent fundus screening with the consent of their families. Infants with severe systemic diseases (e.g., tetralogy of Fallot and phenylketonuria) or other eye diseases (e.g., congenital cataracts and primary congenital glaucoma) or whose mothers had known hereditary diseases were excluded from the study. The study was approved by the ethics committee at the Affiliated Zhongshan Hospital of Sun Yat-sen University (Zhongshan City People's Hospital).

### 2.2. Data Collection

We collected detailed information for each newborn, taking into account general, maternal, obstetric, and neonatal parameters. General parameters included the maternal age, gestational age, infant gender, and birth weight. Maternal parameters included cesarean history, gestational diabetes (GDM), pregnancy-induced hypertension, anemia, thalassemia, placental abruption, premature rupture of membranes, preeclampsia, and eclampsia. The obstetric parameters included mode of delivery (spontaneous vaginal delivery (SVD) versus cesarean section (CS)), use of forceps, postpartum hemorrhage, emergency birth, uterine inertia, duration of the first stage and second stage of labor, birth canal laceration (e.g., cervical laceration, vaginal laceration, and perineal laceration), fetal biparietal diameter and amniotic fluid index (within one week before birth), and pelvic measurements (including the anterior superior iliac spine diameter, iliac spine diameter, sacral shame diameter, and ischial tuberosity diameter). Neonatal parameters included intracranial hemorrhage, cranial hematoma, cord around the fetal neck, neonatal hyperbilirubinemia (NHB), maternal ABO blood group incompatibility, and in vitro fertilization pre-embryo transfer (IVF-ET).

### 2.3. Eye Examination

Eye examinations were conducted within one week of birth (mean = 1.92 ± 2.12 days). All eye examinations, imaging, and readings were performed by a team of experienced retina specialists and nurses. First, the anterior segment was examined by performing the standard swinging flashlight test for the pupillary light reflex. Next, the pupils were dilated with three drops of 1% compound tropicamide eye drops (Santen Pharmaceutical Co. Ltd, Japan) administered ten minutes apart and topical anesthesia (Alcaine eye drops, Alcon) was applied. A child's speculum was used to keep the eyes open and 130° wide-angle digital images were taken with RetCamIII (Clarity Medical Systems, Pleasanton, California, USA). Two experienced reviewers (Zhang Yanli and Lin Yu) independently read the images, and disagreements were resolved by a third senior reviewer (Zhao Qi).

### 2.4. Classifying Degree of Neonatal RH

**Figure 1:**
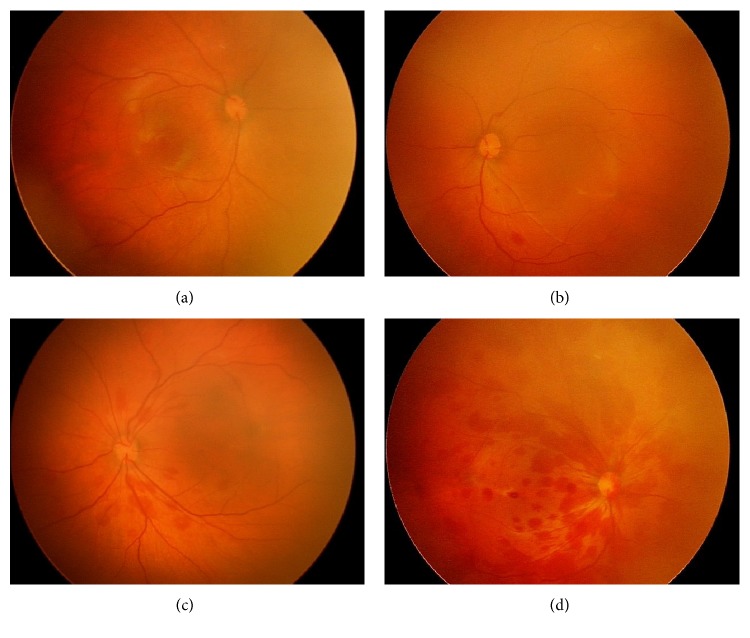
Degree of neonatal retinal hemorrhage. (a) Normal retina. (b) Degree I RH. (c). Degree II RH. (d) Degree III RH.

Based on the Egge et al. [3] classification criteria (Figure 1, from our study), three degrees of neonatal RH were identified, which were degree I: small hemorrhage within a limited range with fine linear bleeding confined to the area around the optic disc; degree II: slightly larger amount of hemorrhage with patchy, flame-shaped bleeding over an area not exceeding the optic disc diameter; and degree III: hemorrhage exceeding the diameter of the optic disc area with flame-shaped bleeding along vessels and macular hemorrhage. Infants without RH were included as controls (group 1), while those with degrees I, II, and III RH were sorted into groups 2, 3, and 4, respectively. When the degree of RH was different in both the eyes, the infant was grouped based on the eye that had more severe bleeding.

### 2.5. Statistical Analysis

Using SPSS 19.0, we conducted univariate logistic regressions comparing differences in the maternal, obstetric, and neonatal factors between newborns with RH (degrees I, II, and III) and those without RH (control group). For factors with *p* values lower than 0.1, we then performed another multinomial logistic regression to remove interfering confounds, and factors with *p* values lower than 0.05 were considered statistically significant.

## 3. Results

### 3.1. Descriptive Statistics of General, Maternal, Obstetric, and Neonatal Parameters in Full-Term Newborns

See Table 1.

### 3.2. Effects of General Factors on the Degree of RH in Full-Term Newborns

A total of 3054 full-term newborns underwent RetCamIII examination; 44 were twins and 3010 were singletons. The male-female ratio was 1.15 : 1, average gestational age was 39.30 ± 1.06 weeks, and mean birth weight was 3220.56 ± 385.51 g. Among the 3054 newborns, 1202 (39.36%) had RH. Of these, 408 (13.36%) were classified as degree I RH, 610 (19.97%) were classified as degree II RH, and 184 (6.03%) were classified as degree III RH. The remaining 1852 newborns (60.64%) did not exhibit RH. A univariate logistic regression showed that the birth weight and newborn's gender were significant factors (*p* < 0.05; see Table 2).

### 3.3. Effects of Maternal, Obstetric, and Neonatal Factors on the Degree of RH in Full-Term Newborns

The results of a univariate logistic regression indicated that the mode of delivery, cesarean history, birth canal laceration, duration of the second stage of labor, cord around the fetal neck, and intracranial hemorrhage had significant effects (*p* < 0.05; see Table 3).

### 3.4. Multivariate Logistic Regression Analysis of Factors Affecting the Degree of RH

Univariate logistic regression results revealed that the newborn's gender, birth weight, intracranial hemorrhage, mode of delivery, cesarean history, maternal age, duration of the first stage of labor, duration of the second stage of labor, emergency birth, premature rupture of membranes, anterior superior iliac spine diameter, birth canal laceration, and cord around the fetal neck correlated with the degree of RH in newborns (*p* < 0.1). These factors were further analyzed using multivariate logistic regression, which showed that the mode of delivery (SVD/CS), duration of the second stage of labor, maternal age, and newborn's intracranial hemorrhage had significant effects on the degree of RH in newborns (*p* < 0.05; see Table 4). Therefore, spontaneous vaginal delivery, prolonged duration of the second stage of labor, advanced maternal age, and newborn's intracranial hemorrhage are factors that aggravate the degree of RH in full-term newborns, while cesarean section is a protective factor against the incidence of RH.

## 4. Discussion

RH is one of the most commonly seen ocular abnormalities in newborns, and it may have a serious impact on the visual function and quality of life of the affected child when it affects the macula. In the recent years, we have witnessed an increase in the detection rate of neonatal RH with the rapid development of neonatal retinal examination methods as well as the advancement of RetCamIII and other emerging technologies, and there are mounting concerns about neonatal retinopathy [4]. As a common form of neonatal abnormality, neonatal RH is increasingly gaining the attention of pediatricians and ophthalmologists.

In this study, the incidence of neonatal RH was 39.36%, which is consistent with the findings from our previous study [5], as well as those of Mao et al. [6] and Liu et al. [7]. In the recent years, researchers have begun studying the risk factors and mechanisms underlying neonatal RH. For instance, Choi et al. [8] proposed a link between RH and pressures on the fetal head in the birth canal, such as compression of the fetal head during delivery causes deformation, which leads to elevated intracranial venous pressure, peripheral vascular congestion, expansion, or rupture, resulting in hemorrhage. Neonatal asphyxia and neonatal hypoxic ischemic encephalopathy can contribute to retinal hypoxia, and rupturing of the lysosomes produced during hypoxia triggers autolysis, leading to retinal capillary endothelial cell necrosis and hemorrhage. In addition, hypoxia can lead to vasospasm, luminal stenosis, vascular endothelial swelling, and increased vascular access, resulting in hemorrhage. The retinal vein dilates during hypoxia, and the increased blood volume and greater blood viscosity causes retinal vasodilation or hemorrhage [9].

The results of this series of studies suggest that SVD is a risk factor for neonatal RH and that cesarean section is a protective factor [5]. Likewise, we found, in the current study, that SVD was associated with increases in the degree of RH, while cesarean section was associated with reductions in the degree of RH [10]. Watts et al. [2] similarly report that, compared with SVD, cesarean section significantly lowers the incidence of neonatal RH. The underlying mechanism may be due to the compression of the fetal head during vaginal delivery. Specifically, the intracranial pressure suddenly increases during vaginal delivery, which is accompanied by a pressure increase in the central retinal vein. At the same time, the scalp veins and intracranial veins dilate due to venous return obstruction. Because vascular walls are thinner in newborns, they rupture easily, resulting in opportunities for RH and a corresponding increase in its severity.

Based on the unilateral logistic regression analysis, we learned that the presence of a cord around the fetal neck is a risk factor for newborn retinal hemorrhage. As the link between the fetus and the placenta, the umbilical cord is the only source of fetal nutrition, and it serves as a channel for material exchange with the mother. A cord around the fetal neck is a common complication in obstetrics [11–13], which may lead to intrauterine growth retardation, neonatal intracranial hemorrhage, asphyxia, and other adverse pregnancy outcomes [14–16]. The mechanism by which the cord around the fetal neck causes retinal hemorrhage includes neonatal hypoxia, vascular wall hypoplasia, and increased vascular permeability, which further leads to abnormal coagulation. With the progression of labor in the delivery process, the fetus first descends gradually as contractions increase, and the umbilical arteriovenous vessels undergo a pulling tension with the cord around the fetal neck, reducing the fetal arterial blood supply, which then leads to short-term ischemia and hypoxia in the blood vessels in the brain, including the retinal artery. At the same time, obstruction of venous return and blood circulation also results in tissue ischemia and hypoxia [17]. When this complication occurs, the neck of the fetus undergoes pulling compression by the umbilical cord. At the same time, blood circulation is obstructed due to venous return obstruction and capillary congestion and dilation. Under the combined effect of these factors, cell metabolism in the retinal peripheral vascular wall is disrupted and the extracellular matrix function is impaired, which eventually leads to telangiectasia, rupture, and increased permeability [18]. However, the results of this study indicated that the relationship between the presence of a cord around the fetal neck and the degree of retinal hemorrhage was not significant. This finding could be because most mothers experiencing this complication choose cesarean section to avoid or reduce compression of the blood vessels in the fetal neck during the delivery process, as well as the intravenous compression from excessive pulling and movement of the umbilical cord itself.

In this study, we found that a prolonged duration of the second stage of labor is a risk factor for increased retinal hemorrhage. During the second stage of labor, the cervix is fully dilated, the fetal head is descended, and contractions are stronger. At the same time, the fetus is prone to fetal intrauterine ischemia and hypoxia due to umbilical cord compression, pulling, and so forth Moreover, hypoxia causes increased reflexivity of acidic metabolites, which excites the vagus nerve. In turn, bowel movement is accelerated, and contamination of the amniotic fluid with meconium discharge may also increase fetal hypoxia. Therefore, the longer the second stage of labor, the more serious the damage to the retinal vein and vascular endothelial cells, causing the former to be a risk factor for increased neonatal retinal hemorrhage [19]. We also found that advanced maternal age is a risk factor for increased retinal hemorrhage. Women of advanced maternal age tend to experience anxiety, depression, and other negative emotions, increasing the risks toward themselves and their fetuses [20]. Such negative emotions and psychological burden can cause uterine inertia and extend the second stage of labor, increasing the degree of retinal hemorrhage.

Through multivariate logistic regression analysis, we found that intracranial hemorrhage is a risk factor for increased retinal hemorrhage. Based on animal experimentation, Kaur and Taylor [21] reported that when intracranial pressure is increased, the retinal vein dilates and venous return is obstructed, causing disc edema and retinal hemorrhage. As such, newborns with serious retinal hemorrhage should be treated as suspects for accompanying intracranial hemorrhage, while newborns with intracranial hemorrhage should undergo fundus examination to exclude the presence of retinal hemorrhage.

There is no uniform standard within China for classifying neonatal RH; most use the internationally recognized classification criteria from Watts et al. and Egge et al. [2, 3], which we adopted in this study. As these criteria were established prior to the advent of wide-angle retinal imaging, this classification system does not take peripheral RH into consideration. With the increasingly popular use of RetCamIII in neonatal fundus screening, these criteria have been found to exhibit certain limitations. Moreover, Egge and Watt's classification criteria focus on the amount of hemorrhage in the retina as a whole, neglecting the potential clinical significance of the impact that macular hemorrhage and the absorption time may have on long-term visual function. Hence, there is a need to investigate more rigorous methods for classifying neonatal RH.

As symptoms such as placenta abruption, eclampsia, cranial hematoma, and related conditions did not surface in all of the different groups of newborns with RH, our data do not represent the full extent of demographic characteristics, and there is currently no way to evaluate their impact on the degree of neonatal RH. Therefore, more centralized, large-scale epidemiological studies should be conducted to help clarify the factors affecting the degree of neonatal RH and to facilitate the effective prevention and treatment of neonatal ocular diseases.

In summary, our results suggest that SVD, prolonged duration of the second stage of labor, advanced maternal age, and neonatal intracranial hemorrhage are risk factors that exacerbate the degree of neonatal RH. Most importantly, close attention to newborns with these risk factors will help with the early detection and timely treatment of visual function abnormalities, improving the quality of life of affected children. It is recommended that clinical obstetricians, neonatologists, and ophthalmologists pay attention to these factors and perform targeted fundus screening on newborns to assist in early intervention efforts.

## Figures and Tables

**Table 1:** Descriptive statistics of clinical parameters in full-term newborns.

	Total (3054)	Group 1 (1202)	Group 2 (408)	Group 3 (610)	Group 4 (184)
Gestational age, mean (min, max)	39.3 (38.6, 40)	39.3 (38.6, 40)	39.3 (38.7, 40.1)	39.3 (38.6, 39.7)	39.4 (38.7, 40)
Birth weight (g), mean (min, max)	3200 (2950, 3480)	3220 (2970, 3500)	3185 (2930, 3432.5)	3090 (2852.5, 3395)	3200 (2970, 3400)
Maternal age (years), mean (min, max)	28 (25, 31)	28 (25, 31)	28 (25, 31)	27.5 (25, 31)	29 (25.8, 32)
Duration of the first stage of labor (h), mean (min, max)	5.4 (3.8, 7.8)	5.7 (3.8, 8)	5.2 (3.8, 7.4)	4.8 (3.4, 6.8)	4.8 (3.3, 6.6)
Duration of the second stage of labor (h), mean (min, max)	0.5 (0.3, 0.8)	0.5 (0.3, 0.9)	0.4 (0.3, 0.7)	0.4 (0.2, 0.7)	0.3 (0.2, 0.7)
Biparietal diameter (mm), mean (min, max)	94 (91, 96)	94 (91, 96)	94 (91, 96)	94 (92, 95)	94 (91, 96)
Amniotic fluid index (mm), mean (min, max)	107 (88, 130)	106 (86, 130)	107.5 (90, 127)	109 (89.3, 129.5)	108 (92, 134.3)
Anterior superior iliac spine diameter, mean (min, max)	24 (24, 25)	24 (24, 25)	24 (24, 25)	24 (24, 25)	24 (24, 25)
Iliac spine diameter, mean (min, max)	27 (27, 27.5)	27 (27, 27.5)	27 (27, 27)	27 (27, 28)	27 (27, 27.1)
Sacral shame diameter, mean (min, max)	19 (19, 19)	19 (19, 19)	19 (18.5, 19)	19 (18.5, 19)	19 (18.5, 19)
Ischial tuberosity diameter, mean (min, max)	8.5 (8, 8.5)	8.5 (8, 8.5)	8.5 (8, 8.5)	8.5 (8, 8.5)	8.5 (8, 8.5)
Gender—male, *n* (%)	1636 (53.6)	1237 (54.9)	212 (52.0)	95 (45.2)	92 (50.0)
Gender—female, *n* (%)	1418 (46.4)	1015 (45.1)	196 (48.0)	115 (54.8)	92 (50.0)
Mode of delivery (SVD), *n* (%)	2117 (69.3)	1363 (60.5)	377 (92.4)	204 (97.1)	173 (94.0)
Mode of delivery (CS), *n* (%)	937 (30.7)	889 (39.5)	31 (7.6)	6 (2.9)	11 (6.0)
Cesarean history, *n* (%)	432 (14.2)	401 (17.8)	17 (4.2)	7 (3.3)	7 (3.8)
Anemia, *n* (%)	126 (4.1)	93 (4.1)	15 (3.7)	6 (2.9)	12 (6.5)
Thalassemia, *n* (%)	102 (3.3)	75 (3.3)	8 (2.0)	10 (4.8)	9 (4.9)
Pregnancy induced hypertension, *n* (%)	48 (1.6)	40 (1.8)	3 (0.7)	2 (1.0)	3 (1.6)
GDM, *n* (%)	217 (7.1)	168 (7.5)	29 (7.1)	11 (5.2)	9 (4.9)
Birth canal laceration, *n* (%)	1328 (43.5)	836 (37.1)	240 (58.8)	134 (63.8)	118 (64.1)
Preeclampsia, *n* (%)	71 (2.3)	57 (2.5)	9 (2.2)	2 (0.9)	3 (1.6)
Eclampsia, *n* (%)	0 (0)	0 (0)	0 (0)	0 (0)	0 (0)
Uterine inertia, *n* (%)	206 (6.7)	155 (6.9)	25 (6.1)	17 (8.1)	9 (4.9)
Emergency birth, *n* (%)	82 (2.7)	53 (2.3)	13 (3.2)	11 (5.2)	5 (2.7)
Polyhydramnios, *n* (%)	18 (0.6)	14 (0.6)	2 (0.5)	1 (0.5)	1 (0.5)
Oligohydramnios, *n* (%)	90 (2.9)	69 (3.1)	12 (2.9)	7 (3.3)	2 (1.1)
Postpartum hemorrhage, *n* (%)	74 (2.4)	56 (2.5)	12 (2.9)	5 (2.4)	1 (0.5)
Premature rupture of membranes, *n* (%)	535 (17.5)	411 (18.2)	58 (14.2)	42 (20.0)	24 (13.0)
Placental abruption, *n* (%)	7 (0.2)	5 (0.2)	2 (0.5)	0 (0)	0 (0)
Cord around the fetal neck, *n* (%)	866 (28.4)	613 (27.2)	130 (31.9)	64 (30.5)	59 (32.1)
Intracranial hemorrhage, *n* (%)	18 (0.6)	4 (0.2)	10 (2.5)	0 (0)	4 (2.2)
Cranial hematoma, *n* (%)	3 (0.1)	3 (0.1)	0 (0)	0 (0)	0 (0)
NHB, *n* (%)	841 (27.5)	604 (26.8)	116 (28.4)	66 (31.4)	55 (29.9)
Maternal ABO blood group incompatibility, *n* (%)	34 (1.1)	26 (1.1)	4 (1.0)	2 (0.9)	2 (1.1)
Forceps, *n* (%)	30 (0.1)	21 (0.9)	5 (1.2)	2 (0.9)	2 (1.1)
IVF-ET, *n* (%)	25 (0.8)	22 (1.0)	3 (0.7)	0 (0)	0 (0)

Group 1 = control; Group 2 = degree I RH; Group 3 = degree II RH; Group 4 = degree III RH.

**Table 2:** Univariate logistic regression analysis of general factors affecting the degree of RH in full-term newborns.

General factors	*OR*	95% CI		*p*
		Lower	Upper	
Maternal age	0.984	0.967	1.002	0.079
Birth weight	1.000	1.000	1.000	<0.001^a^
Gender	1.239	1.056	1.453	0.008^a^
Gestational age	1.009	0.935	1.089	0.817

^a^Statistically significant.

**Table 3:** Univariate logistic regression analysis of maternal, obstetric, and neonatal factors on the degree of RH in full-term newborns.

	*OR*	95% CI		*p*
		Upper	Lower	
Maternal factors				
Cesarean history	0.186	0.128	0.271	<0.001^a^
Anemia	1.037	0.693	1.550	0.861
Thalassemia	1.096	0.702	1.710	0.687
Pregnancy-induced hypertension	0.581	0.271	1.246	0.163
GDM	0.789	0.570	1.093	0.154
Preeclampsia	0.669	0.373	1.202	0.179
Premature rupture of membranes	0.827	0.666	1.028	0.087
Placental abruption	0.940	0.192	4.593	0.939
Obstetric factors				
Mode of delivery (SVD/CS)	0.097	0.072	0.132	<0.001^a^
Birth canal laceration	2.671	2.268	3.146	<0.001^a^
Uterine inertia	0.917	0.664	1.267	0.599
Emergency birth	1.539	0.985	2.406	0.058
Polyhydramnios	0.809	0.268	2.443	0.706
Oligohydramnios	0.825	0.506	1.344	0.439
Postpartum hemorrhage	0.846	0.499	1.434	0.534
Duration of the first stage of labor	0.976	0.950	1.003	0.080
Duration of the second stage of labor	0.654	0.540	0.792	<0.001^a^
Amniotic fluid index	1.001	0.999	1.003	0.285
Forceps	1.179	0.546	2.546	0.675
Anterior superior iliac spine diameter	0.923	0.842	1.011	0.084
Iliac spine diameter	1.011	0.931	1.097	0.799
Sacral shame diameter	0.909	0.799	1.034	0.147
Ischial tuberosity diameter	0.969	0.880	1.067	0.517
BPD	1.004	0.985	1.023	0.674
Neonatal factors				
Cord around the fetal neck	1.223	1.029	1.454	0.022^a^
Intracranial hemorrhage	4.932	2.263	10.75	<0.001^a^
NHB	1.150	0.965	1.371	0.119
Maternal ABO blood group incompatibility	0.870	0.395	1.915	0.729
IVF-ET	0.359	0.108	1.194	0.095

^a^Statistically significant.

**Table 4:** Multivariate logistic regression analysis of risk factors for RH in full-term newborns.

Risk factors	*OR*	95% CI		*p*
		Lower	Upper	
Maternal age	1.028	1.007	1.049	0.008^a^
Duration of the second stage of labor	0.689	0.569	0.835	<0.001^a^
Intracranial hemorrhage	3.505	1.598	7.686	0.002^a^
Gender	1.170	0.982	1.394	0.079
Mode of delivery (SVD/CS)	1.463	1.459	1.468	<0.001^a^
Cord around the fetal neck	1.118	0.926	1.351	0.247

^a^Statistically significant.
